# On Dysentery, Its Forms and Consequences, in Warm Climates, Especially in India

**Published:** 1831

**Authors:** 


### ON DYSENTERY, ITS FORMS AND CONSEQUENCES, IN WARM CLIMATES, ESPECIALLY IN INDIA. By *James Annesley*, Esq.

[Vol. II. of Diseases of India.]

WE have reason to lament, in common with the profession generally, but more especially those who are scattered over our Indian possessions, that the inestimable work of Mr. Annesley was not published separately from the plates, and consequently in a form that would have insured these expensive volumes an extended circulation. Had it not been for the medium of this Journal, the volumes might almost be said to be buried; but we have endeavoured to diffuse a great portion of the valuable materials with which they are fraught, through both hemispheres, and shall not cease till our object is accomplished. We have brought our analysis up to about the third of the second volume, where the important subject of dysentery is taken up by the experienced author. We need not advert to the importance of a malady which forms the prominent feature of tropical diseases, and carries off more Europeans than any other form of disease whatever. The author treats first of the simpler or less complicated forms of dysentery—then of that variety which is characterized by attendant hepatic disorder—and, lastly, of the chronic forms, and on scorbutic dysentery.

### SECT. I. ACUTE UNCOMPLICATED DYSENTERY.

Collections of excrementitious matters, Mr. A. thinks, form one of the earliest pathological states which give rise to acute dysentery, by irritating the mucous membrane, and inducing inflammation, followed by ulceration and even sphacelation, if the case be neglected.

"In a great many cases, this form of dysentery is preceded by a constipated state of the bowels, often of long duration, especially among persons who have recently arrived in India. To this condition frequently supervenes mucous diarrhœa, attended with pains of the abdomen, coming on at intervals, and generally preceding the alvine evacuations. This form of diarrhœa may continue for two or three days, passing gradually into dysentery, with all the characteristic signs of the disease. In a few instances, especially when the evacuations are copious, the diarrhœa subsides, and the patient recovers without experiencing, at least for that time, a true dysenteric attack. This result seems to arise from the irritation produced upon the mucous surface of the large bowels by the fæcal accumulations having subsided, in consequence of the irritating matters having been removed, and of the copious secretion which had taken place.

Frequently, the dysenteric symptoms are present from the first hour at which the patient complains, the stools being then scanty, mucous, streaked with blood, and attended with abdominal pain and tenesmus. In cases of this nature, the increased action of the muscular coats of the bowel, especially about the sigmoid flexure and rectum, prevents the passage of the fæcal collections through their canal, and, in many cases, occasions a complete obstruction, little passing away but the perfectly fluid secretions. In cases of this description, if the disease be not early subdued by very decided treatment, sloughing of the mucous coat often takes place, followed by involuntary motions, when the fæcal accumulations at last come away, such parts of them, at least, as have been dissolved being washed off by the watery secretions poured out from the irritated vessels of the inflamed surface." 153.

Believing this form of dysentery then to be essentially an inflammatory disease, whatever its cause, our author takes occasion here to dissent from the late Mr. Bampfield in respect to the subdivisions of the disease which that gentleman adopted. "The mild, the severe, and the inflammatory varieties which he has marked out, are, in our opinion, nothing more than varying degrees of the same, or nearly similar pathological states, proceeding from the extent to which the inflammatory action may have *supervened*, from the susceptibility of the system to sympathise with the local disease, and from the peculiarity of individual constitution. There is no line of demarkation by which these varieties can be separated from each other in practice."

Viewing the acute and uncomplicated form of dysentery, then, as an inflammation limited chiefly to the cæcum, colon, and rectum, Mr. A. proceeds to paint its various degrees of intensity, as presented in practice among recently arrived Europeans, in older residents, and among the natives. We shall here introduce the description which he draws.

"Simple dysentery, in its least severe forms, generally commences with frequent calls to stool, the motions being scanty, mucous, gelatinous, streaked with blood, and accompanied with pain and tenesmus. At first the pain seems chiefly limited to the rectum, occasional griping pains being only felt in the abdomen. The tongue is often but little affected, farther than being white and loaded; the pulse sometimes at the beginning not materially accelerated, but it generally soon becomes affected to an extent varying according to the habit of the patient and severity of the disease. If the disorder be not subdued in this early stage, all the symptoms become more acute; the pain in the abdomen increases in severity and is more constant, yet, in many cases, little or no pain is complained of, excepting at the time when the patient is passing a motion, although the stools are of the most morbid character, and the disease altogether of the most severe form. This, however, ought not to be imputed to the absence of inflammatory action; for the mucous surface of the cæcum, colon, and rectum, may be inflamed, and, indeed, in a state of ulceration, and yet but little uneasiness, even upon firm pressure of the abdomen, is apparently felt. This seems to be owing to the varying degree of excitability and sensibility with which the human frame is endowed, and, perhaps, to some modification in the condition of the diseased parts, beyond the detection of our unaided senses. Yet, in many cases, where pain is either entirely absent, or but little complained of, a sense of heat in the abdomen, especially in the course of the colon, is very generally felt. When this symptom is present, it ought always to be recognised as indicating the existence of inflammation of the mucous surface of the bowel. A similar inference ought also to be deduced from a sense of soreness in the abdomen. This symptom is very often present in all the stages of the disease, and always indicates great irritation of the mucous surface. It frequently accompanies the sensation of heat, or supervenes to that symptom. (See the case of Thomas Morgan, at the end of this section.)

As long as the disease is limited to the mucous lining of the large bowels, the patient seldom feels more than a sense of heat, or a dull aching pain, not increased on pressure, which he usually describes as being heavy, and shooting at times through the whole abdomen: but when the cæcum is minutely examined, pain, to a greater or less extent, is always felt, and, perhaps, some degree of fulness, even when pressure over the transverse arch of the colon occasions no uneasiness. If the left side of the abdomen, beneath the ribs, be grasped in the hand, so as to embrace the descending colon and sigmoid flexure, pain is sometimes felt, but not always; but when the right side is similarly grasped, so as to press upon the cæcum in opposite directions, then pain is almost always complained of. If the practitioner takes the patient's report without further examination, in cases of this description, he will often be misled.

As the disease advances, the stools usually become still more frequent, the tenesmus more severe, the discharges of blood greater and often more intimately mixed with the matters evacuated, which gradually pass from a mucous, slimy, and gelatinous character, to a more watery appearance, of a dark colour, with a muddy solution of feculent matters, and sometimes with considerable discharges of fæces. The urine is now, and often early in the disease, of a high colour, voided frequently, and attended with scalding. Sometimes complete strangury is present: this is owing to the intimate connexion subsisting between the urinary organs and the seat of disorder. The tongue becomes more loaded and excited; the pulse more accelerated; and the skin harsh, hot, and dry. Tormina also, and the straining, increase; the calls to evacuation become more incessant, especially during the night, when the general febrile symptoms also are augmented.

When the straining and tenesmus are very urgent, we may then consider the rectum to be very remarkably inflamed: indeed, we know not of an instance where such a state was not evident when these symptoms were present. If tenesmus be very severe, in any particular instance, and if the patient presents but little abdominal fulness or tension,—if he complains but little of tormina, or of heat and soreness in the abdomen,—if he can bear pressure without uneasiness being produced about the region of the cæcum and sigmoid flexure of the colon,—we may then consider that disease is chiefly seated in the rectum, and that the large bowel is comparatively exempt, or at least much less affected than the rectum. But although this inference may be drawn, especially if there be little constitutional disturbance present, we ought not to depend upon it with certainty, and we should never allow it to seduce us into the adoption of weak measures of cure.

We have often seen the most extensive ulceration in the cæcum and colon, and yet the patient had not complained of tenesmus, the rectum having been comparatively sound. (See the case of Thomas Dunn, detailed at the end of this section.) And we have seen tenesmus to a great and distressing degree, the colon, throughout its extent, being, upon *post mortem* examination, found little disordered, and the disease confined to the rectum. From these circumstances, therefore, we have during the latter years of our practice, especially when tenesmus has been urgent, considered it merely as characteristic of disease of the rectum, although frequently an attendant upon dysentery, and treated it accordingly, whether it arose at the commencement of the disease, or during the advanced stages." 157.

We shall not follow the author in his remarks on dysentery as it affects the natives of India. In them, as might be expected, the disease assumes lower or more asthenic form, though the appearances after death, are nearly the same in both classes.

In simple acute dysentery, the number of calls to evacuate the bowels varies from ten or twelve, to thirty or forty times in the twenty-four hours—many of the stools being merely a small quantity of mucus and blood—some of them more copious, and consisting of various morbid secretions as well as excretions. These fluid motions rapidly exhaust the strength of the patient. These watery evacuations, Mr. A. thinks, are indicative of "the lodgment of acrid matters in the bowels requiring to be removed by purgatives at the commencement of the attack." Of this we have some doubts.

The author indeed tells us immediately afterwards, that, "in some cases, the disease seems to commence in the rectum, &c." The appetite is sometimes so little impaired in simple dysentery, that ulceration of the intestines has taken place before food was loathed. But food generally produces great uneasiness in the line of the bowels, even where the appetite is unimpaired. After a number of minute and rather tiresome observations on various phenomena presented by the disease in question, Mr. A. proceeds thus :—

"Besides the appearances of the stools already pointed out, there are others which are less constant, and which deserve notice. The evacuations are sometimes of a singularly variegated hue, consisting of a glairy mucus, mixed with a greenish, gelatinous substance, sometimes with pure bile, at other times with a muco-purulent matter, with large pieces of albuminous-like concretions formed upon the internal surface of the bowel and afterwards detached, and either with streaks of fluid blood, or with dark coagula, more or less intimately mixed with the other matters discharged. Blood is occasionally evacuated in very large quantities, fluid, and distinct from the other matters composing the evacuation: it then flows from the lower parts of the large bowels. When consisting of coagula, and of dark, grumous clots intimately mixed with the discharges, we may consider it as having proceeded from the upper parts of the colon, or from the cæcum itself. The discharge of pure blood sometimes takes place early in the disease, and continues to its termination in death, (see James's case); but this intestinal hæmorrhage is seldom of a florid hue: it most frequently presents the venous character, and occasionally a dark-brown, muddy appearance, mixed intimately with watery, feculent, and offensive dejections. The copious sanguineous discharge may or may not proceed from an ulcerated surface. We believe that it most frequently exudes from the irritated mucous surface, and that the latter description of discharge is characteristic of ulceration, and occurs most frequently in persons who have neglected the state of their bowels, or who have indulged in the intoxicating liquors of India, which are so destructive to soldiers." 163.

The mucous membrane of the rectum or colon is sometimes detached, in the latter stages of the disease, and discharged per anum in a tubular form. Most frequently, however, these membrane-looking tubes are exudations of coagulable lymph thrown out upon the inflamed surface of the bowel. When dysentery has advanced to the most unfavourable stage, the stools are streaked with purulent or sanious matters, evidently proceeding from ulceration of the bowels. We pass over a great mass of cases, and tedious details that are spun out to an immeasurable length, when we come to a section on—

### Hepatic Dysentery.

This is a form of the disease remarkably prevalent in India; consequently, its nature and treatment are of the utmost importance. This species of dysentery assumes various forms—is sometimes acute, sometimes sub-acute—or chronic. The more acute forms are generally accompanied by an affection of the liver, and a highly vitiated condition of the biliary secretion—while the chronic forms are attended by abscess and other organic changes of that viscus. Mr. Annesley does not attempt an explanation of this connexion of hepatitis and dysentery, nor to the precedency of one in respect to the other. The two phenomena, indeed, are often so coeval, that it is difficult, or rather impossible to ascertain the priority of one or the other. While wading through many pages of heavy quarto, presenting loads of the most insipid verbiage, and where the everlasting word "*supervene*" occurs in almost every sentence, we have been unable to find a passage to extract, or matter for condensation in our own language. What could infatuate Mr. Annesley to dilute his facts with such an ocean of words?

"In many cases of hepatic as well as of simple dysentery, the patient presents, for a day or two, many of the symptoms particularized in the section on the premonitory signs of disease contained in the First Volume of the Work, page 209. But this is not uniformly the case. The countenance is often pale, the skin cold, with horripilation, sickness, and loss of appetite, and a disordered, costive, and irritative state of the bowels. The patient often, at the same time, complains of a sense of chilliness, coldness or uneasiness in the back and lumbar region, running down the sacrum, sometimes as far as the anus, with griping pains through the abdomen. These symptoms, however, seldom fall under the observation of the practitioner, unless he makes it a duty to inquire particularly into the condition of the men under his charge during health; for it is not generally until the phenomena pathognomonic of simple or complicated dysentery are fully developed, that medical advice is sought after. In those cases of hepatic dysentery in which the complication is immediate, or disease nearly coeval in both organs, the premonitory signs now noticed are often well marked, the griping pains extend through the abdomen and hypochondria, and are sometimes attended with vomiting, a sense of fulness and oppression at the præcordia, lowness of spirits, and slight dyspnœa.

At the commencement of this particular form of the disease, and generally following the above symptoms, the alvine dejections become frequent, and at first are usually copious, but morbid, both in colour, consistence, and odour. At this period they are very seldom either mucous or bloody, but they are generally very dark, crude, and offensive. As the disease advances, they vary daily, but are generally green, bottle-green, greenish brown or black, mixed with venous blood; sometimes slimy and watery, with a greenish frothy slime on the surface; rarely clay-coloured, and not infrequently, especially in the advanced stage of the worst cases, reddish brown, ochre-like, or consisting chiefly of water, with blood more or less intimately diffused through it. The motions vary in frequency and in character, according to the stage of the disease and the treatment adopted. There is generally urgent tenesmus present, with scalding of the anus, and often *prolapsus ani*. The calls to stool are more frequent during the night, and attended with more or less irritative fever and restlessness. Sometimes the blood is so very intimately mixed with the other matters forming the alvine evacuations, that it must have proceeded either from the superior portions of the alimentary canal, or from the liver itself. But this is an appearance observed chiefly in the far advanced stage of the disease, when also the evacuations often resemble the washings of raw meat, and present nearly similar characters to those marking the last period of the simple form of the disease. The urine is generally in very small quantity, high-coloured, muddy, and evacuated usually with pain and difficulty.

In addition to this state of the alvine excretions, the patient generally complains of a fixed pain, weight, or uneasiness, in the pit of the stomach, increased on pressure, and frequently extending to the right hypochondrium, and beneath the right scapula. There are usually also present tension, and a sense of pressure at the hypochondrium, with anxiety at the præcordia, fits of dyspnœa, occasionally pain in the right shoulder, or in the chest, with a dry, teasing cough, headach, giddiness, sickness at stomach, sometimes vomiting, and great depression of spirits. The pulse is generally accelerated and irritable, especially towards night.

The appearance of the tongue is various in different stages of the disease, and in different cases: in the early stages it is generally white, excited, and covered with a yellowish fur. As the disease advances, the tongue either becomes dry, clean, smooth, red, and lobulated, or excited, dry, and covered at the root particularly with a dark crust. The skin is sometimes dry, harsh and of a dirty appearance; occasionally it is covered with a greasy perspiration, and copious sweats often occur through the advanced periods of the disease. There are also frequent thirst, and great desire of cold fluids. In other respects, the progress of hepatic dysentery is much the same as the simple form of disease already described; but it presents, in general, a greater range or variety of phenomena in different cases, and even in the same case, in different stages of the malady." 209.

In the more chronic examples of hepatic dysentery, as connected with abscess, the matter not seldom finds its way into the alimentary canal, through adhesions previously formed—or perhaps through the ducts themselves.

"The symptoms of the chronic forms of hepatic dysentery are more mild: tormina and tenesmus are not severe, if at all complained of. Little or no pain is felt, even upon pressure, in the course of the colon; but the alvine evacuations are always more or less unnatural, and present appearances either of a morbid state of the bile, or of a deficient or obstructed secretion of this fluid. The calls to stool are also not so frequent as in the acute cases; but there are present great debility, depression of spirits, and sinking of the powers of life, particularly in those who have been addicted to intoxication.

In cases of hepatic dysentery, a dirty appearance of the skin, sallow cast of countenance, attended with an expression of anxiety and great depression of the spirits, are very generally present, and may often be relied upon as evincing disease of the liver, even although pain, weight, and tension at the epigastrium, præcordia, right thorax, and hypochondrium, may be wanting. The presence, however, of these latter signs, in addition to the former, and to the symptoms described as characterising the progress of simple dysentery, is distinctive of the associated disease of both organs." 211.

Under the head of Etiology, Mr. Annesley presents us with some documents indicative of the terrible ravages which dysentery occasions among European sojourners in tropical climates. Thus we find that, during a series of years, when the troops were not actively employed in the field, the annual rate of nominal admissions in hospitals (Bengal army) was 35 per cent. of the effective strength, while the annual rate of mortality was $5\frac{1}{2}$ per cent. in the nominal admissions. In the Madras army the rate of mortality was 8 per cent. though the rate of admission was only 27 per cent. Malaria and atmospheric vicissitudes are looked upon, and, no doubt, with justice, by Mr. A. as the chief agents in the production of dysentery, with intemperance and some other causes as auxiliaries. We must pass over a great mass of observations on the etiology of dysentery, which might be useful in an elementary lecture on the subject, but would be misplaced here. They are deserving of attention among those who reside in the East, though few of them can have access to the work, on account of its price and size. We must now proceed to a subject interesting to all classes of our readers.

### POST-MORTEM APPEARANCES.

In those cases where the termination is rapidly fatal, sphacelation of the mucous membrane of the colon is generally observed, with portions of the same inflamed, or ulcerated. Without insisting that inflammation is the primary link in the morbid chain, Mr. Annesley maintains that it is a sine qua non in the complete establishment of the disease.

"It is extremely probable that the inflammatory action has been occasioned by some irritating cause lodged in the *primæ viæ*, inducing simple irritation, in the first instance, of the capillary vessels and exhalants of the mucous coat of the large bowels, or by other causes acting upon the body from without, and producing a determination of the circulating fluids to the same situation, and a similar condition of the vessels; but in whatever way it may originate, there can be no doubt that inflammatory action is, in the acute form of the disease especially, almost coeval with the dysenteric character of the stools; and the treatment which is founded upon this view will generally be the most successful in combating this disease as it is observed in warm climates. But whether dysentery originates in simple irritation, attended with increased exhalation, and terminating in acute inflammation of the mucous surface of the large bowels, or consists of inflammation of this surface from the commencement of the disease, is a question which cannot be solved by *post-mortem* examinations. Both these pathological states may be present in different cases, and may depend upon the causes producing the disease, and the constitution of the individual affected: from their nature they may be expected to produce analogous symptoms, and such as we observe generally to characterise the commencement of dysenteric affections.

Upon opening the abdomen of fatal dysenteric cases, the first object which generally attracts attention is the state of the omentum. This part is frequently inflamed, owing evidently to the extension of the inflammatory action to the peritoneal surface of the large bowels, and thence to this part. Sometimes it adheres, through the medium of coagulable lymph, to the more superficial convolutions of the bowels, at other times to the anterior part of the brim of the pelvis, or even to both: more frequently it is drawn up irregularly to the arch of the colon, and occasionally it seems wrapt close up to this part of the large bowel. Sometimes it is drawn to one side, and adheres both to the colon and to the abdominal parietes. These appearances are the more marked, if the ulcerations in the large bowels, which we shall have immediately to describe, have perforated the bowel, so as to occasion the extravasation of its contents into the peritoneal cavity, thereby producing general peritonitis; and when the dysentery has been complicated with hepatic disease.

With respect to the external appearances of the large bowels, it will be necessary to premise a few remarks before we proceed to describe the state of their internal surface. Sometimes these viscera present no external marks of disease to a superficial observation, and yet they will be found extensively disorganised when laid open. We suppose that it has been owing to their apparently healthy condition externally, that we so frequently have been furnished with accounts of a natural state of the large bowels having been observed in dissections of fatal cases of dysentery. The inference, to our minds, upon reading such accounts, is, that the individuals who furnished them have not inspected the seat of the disease which they had been attempting to investigate. Even when the colon is not remarkably diseased in its external surface, it generally presents one or more of the following states:—It is usually more or less distended with flatus. The colour of its surface is various in different cases, and the shade different in different parts of the same bowel. Upon grasping the viscus, and running the fingers along it, a different feeling is communicated to the touch in distinct parts of it: at one place it is thickened and doughy, in another, thin and membranous. In one part the general shade of colour externally is a bluish-grey; in another, a greenish-blue: in one case it is verging to purple; in another it is pink: sometimes it is obviously inflamed in its serous covering, the capillaries distended with blood, running in all directions, and forming a close reticulum in its surface; occasionally the colour of the surface is quite natural, and the peritoneal covering possessed of its natural diaphanous appearance.

The shades of colour presented by the cæcum and colon exernally, although frequently depending upon, or having some relation to, the states of the internal coats of these viscera, yet sometimes have no such dependence: thus we have observed, in cases where the peritoneal surface was the most pale, the internal or mucous surface of the bowel was most deeply diseased, of the darkest colour, and either sphacelated or extensively ulcerated. In other cases, where this viscus was externally of the deepest colour, varying in some parts from a brick-red to a reddish-brown or deep purple, the internal surface has sometimes presented less than usual marks of disease in those situations. Hence, although the colour of the bowel externally may frequently depend upon the state of disorganization existing internally, yet no such connexion should be necessarily expected.

Displacements, elongations, and unnatural convolutions of the colon, are not infrequently observed in dissection of dysenteric cases. These have been already noticed; but we may further observe, that they are generally connected with some degree of relaxation of the longitudinal bundles of fibres which draw the colon into a sacculated form when in a state of contraction, so that the bowel in those states seldom presents many of those deep circular folds which form its cells; or they exist only in a small degree. In the majority of cases wherein displacements or elongations of the colon have been remarked by us, its peritoneal surface has been inflamed in parts, particularly that portion which was displaced. This is shewn in several of the Plates, where coagulable lymph may be seen covering the displaced portions, and connecting their surfaces either to each other or to adjoining parts, and sometimes to both. Amongst the most frequent displacements of the colon remarked, are,—first, a loop of the sigmoid flexure descending low into the pelvis, close to, sometimes adhering to, the urinary bladder and rectum, and explaining the disorder of the urinary function remarked through the progress of the disease; second, the descent of the transverse arch of the colon, generally towards the right side, nearly as low as the pubes, as represented in the Plates." 259.

Sometimes the cæcum and colon are distended with fetid flatus, and the caliber of the bowel every where increased. In many cases, however, the distentions are partial, and there are many constrictions. The constricted portions are sometimes very limited, and appear as if a ligature had been tied round the gut—in other cases they occupy a considerable extent.

"The constrictions in some cases seem to be chiefly the result of a spasmodic action of the circular fibres of the part affected, owing to the irritation and inflammation of the internal surface. In other cases they seem to be of a more permanent nature; although most probably at their commencement they were the consequence of spasm. When the parts contracted are also found externally inflamed, thickened, and hardened, and in a semi-cartilaginous state, as they frequently are in the more chronic cases, their nature cannot be mistaken; and they must be viewed as one of the results of a slower state of inflammatory action, or of acute inflammation terminating in the chronic form; they are also very often the effect of repeated attacks of the disease. Sometimes the constricted portions of the bowel are remarkably inflamed externally, and occasionally they present in the peritoneal surface no very evident appearance of inflammatory action; although, internally, both inflammation and its consequences are present to a great extent. The narrow constrictions, as if from a ligature, are those which least frequently offer an inflamed appearance externally.

When the constrictions tend nearly to efface the canal of the bowel, the part above is usually much distended, and in some cases the coats of the distended portion are lacerated, and the contents of the bowel effused into the peritoneal cavity. The laceration seldom takes place in a sound part of the bowel; it generally occurs, or at least commences, in a part which has been ulcerated internally, and softened by the existing inflammation. The laceration of the distended part of the bowel is mostly soon followed by the death of the patient, but seldom before evidences of general inflammation of the peritoneal surfaces have been produced by the effused matters, and the bowels are glued together by coagulable lymph; and albuminous exudations, with a turbid serum, are poured out into the abdominal cavity. Sometimes the lacerated portion of the bowel is situated below a constricted part. When this is the case, there is always found a still more constricted portion below the laceration, which is situated in a more or less distended part of the bowel; although, after the laceration has taken place, the extent of the previous distension cannot be ascertained. In addition to the contractions and constrictions of the colon, the parts thus diseased may be still farther deranged: they may be very closely adhering to adjoining viscera, or pressed upon by parts morbidly distended; or they may form very sharp turns and convolutions, tending still further to obstruct their canals; or they may be encumbered by large effusions of coagulable lymph upon their external surface, forming bands or artificial ligatures in the processes of condensation and adventitious organisation, which these effusions often experience when life is prolonged for any considerable time after they first take place. Similar appearances are also observed in respect of the distended parts of the bowels, as shewn by several of the Plates accompanying this Volume." 261.

When the inflammation of the mucous membrane has proceeded so far as to implicate the peritoneal covering, post-mortem examination discloses great vascularity of the latter tunic, sometimes amounting to a purplish hue, with agglutination of the adjoining surfaces, and of the intestines to the liver and other contiguous organs. We now revert back to the state of the internal surfaces.

"In almost all cases of ulceration of the large bowels, the parts ulcerated are softened or more friable, so that they are readily torn upon forcible extension; and if the parts situated between the ulcerations be inflamed, as they generally are, they are also lacerated with ease. Want of the cohesion characteristic of healthy textures seems to be generally present in nearly all instances of the disease accompanied with acute inflammation, or any of its consequences.

Besides the general appearances characteristic of inflammatory action, or resulting from this state, which we have now described, the coats of the large bowels seem much thickened. This is particularly observable in the subacute and chronic cases of the disease. A certain degree of tumefaction or fulness of the inner coats of these bowels seems to depend upon the inflammatory state, and to arise from the general injection of the vessels, and effusion of fluid in the cellular tissue connecting their various coats. A thickened condition of the large bowels is, however, not uniformly remarked: in some few cases their parietes seem thinner than they are even in the healthy state, and are, at the same time, ulcerated to a greater or less extent. Occasionally, one part of the viscus is evidently thinner than natural; whilst another portion is much thickened and as if corrugated, as in Plate XXXIV. fig. 1.

The colour of the internal surface of the large bowels varies very much in different cases, as well as in the same case. In some it is of a very deep red, streaked transversely, and dotted in parts with a darker tint; the edges of the deeper ulcers, and the centres of those in the incipient stages, being of a still darker colour. Sometimes, intervening between large portions of deeply inflamed and ulcerated colon, the mucous surface presents a pale, greenish-yellow hue, with or without small specks of ulceration, as in Plate XXXVIII. Occasionally, the intensely red colour is variegated by the different shades presented by the slight duplicatures and corrugations of the mucous surface of the bowel, and gradually passes into a yellowish or vermillion-red, and thence into a darker shade, indicating the transition to the sphacelated state, as shewn in Plate XXXIII. In some cases, nearly the whole of the mucous surface of the cæcum and colon is of a greenish hue, and presenting every depth of shade, from a pale grass-green to an olive colour; in some parts the deeper shades of green are interspersed with patches of a fine rose colour: in these latter the mucous tissue possesses its natural organisation, whilst, in the former, its cohesion and structure are entirely destroyed, and it is in all respects in the first stage of gangrene." 266.

Our author remarks that, in the complication of hepatic disease with dysentery, in hot climates, "the association of structural changes of the liver with disorganization of the large bowels is constantly observed." It would be very strange were it otherwise. Mr. A. admits, what had been inferred by writers long before him, that the vitiated secretions from the liver are probably a material agent in exciting inflammatory action in the intestines.

### TREATMENT OF DYSENTERY.

The rapid march of dysentery in hot climates requires rapid and active treatment; and in no disease is the utility of remedies, when early and judiciously employed, more conspicuous than in this disease. Unlike many maladies of temperate climates, dysentery is hardly capable of natural solution. On the contrary, it naturally tends to disorganization, if neglected.

The first indication, in uncomplicated dysentery, is to empty the bowels, and the second to guard against inflammation. The other indications are considered as subordinate.

"If the patient comes under our care when the premonitory symptoms of disorder are present,—when the bowels are first disordered, and he complains of chills, followed by slight flushes, coldness of the back and loins, &c.,—the exhibition of an ipecacuanha emetic, followed in a few hours by twenty grains of calomel, and this in two or three hours more by a purging draught and an enema, is often of the greatest benefit. At this time also the warm bath is of great service, by determining the circulation to the external surface of the body and taking off spasm, whilst the evacuating remedies directed to the *primæ viæ* unload it of those accumulations which are so frequently instrumental in producing the disease.

When acute dysentery is fully developed, and the patient complains of a sense of heat, burning, soreness, pain, tormina, &c. then depletion is absolutely requisite, and the sooner it is employed after the supervention of those symptoms, the more likely is advantage to be procured from it. The above symptoms are sufficient of themselves to require its adoption, if the pulse be but little accelerated and the patient not plethoric; and if he have been resident for a considerable time in the climate, local depletions, followed by hot fomentations or warm poulticing, when the leech-bites have ceased to bleed, will generally be sufficient. Depletion to a great extent, or at least to a sufficient extent, may be practised in this way. But if the patient has recently arrived from Europe, if he be of a full habit, if the pulse be full, hard, and irritable, if the tormina be violent, and pain fixed and increased on pressure,—a full blood-letting from the arm should always precede the application of leeches to the abdomen." 273.

The repetitions of bloodletting must depend on the judgment of the practitioner. In addition to depletion, general or local, Mr. A. endeavours to procure free evacuations from the bowels. "With this view, 20 grains of calomel, combined with one or two of opium, should follow the first depletion, and, a few hours afterwards, a purging draught, assisted by an injection, should be administered. The previous exhibition of the calomel and opium allays the irritability of the stomach, if this symptom be present, removes spasm, and prepares the morbid secretions of the liver and bowels for removal by means of the purgatives which are to follow." By the above passage, it will be seen that the plan laid down twenty or thirty years ago by writers on tropical climates, and by the Editor of this Journal in particular, continues to be the standard practice up to this hour. The above plan is almost verbatim et literatim what was acted on during the late war in India, and with the best success. We do not quite agree with Mr. Annesley, however, in his inveterate desire for purgation in dysentery; because we are very far from believing that the retention of fæcal matters in the cells of the colon constitutes the sole, or even the principal cause of the tormina and other phenomena of dysentery. Nevertheless, the plan pursued by our author will be generally successful; because the exhibition of the calomel and opium previously to the purgative, corrects any bad effects that might result from the purgative plan if pursued by itself. Tepid and warm bathing, after the antiphlogistic measures, is an important auxiliary—as are blisters and local fomentations to the abdomen.

"In the far advanced stage of the disease, after the above measures have been employed without deriving from them those advantages which they are calculated to afford, and generally do afford; or when the patient has been neglected or injudiciously treated at the commencement of the malady, the existence of ulceration of the large bowels, either in its incipient or farther-advanced stages, should be dreaded; but we ought not on that account entirely to despair of the recovery of the patient, although an unfavourable termination is more likely to supervene. We have known many cases of recovery wherein the symptoms clearly indicated the existence of ulceration; and even after large portions of the mucous surface of the large bowel had been detached and evacuated with the discharges. Until unequivocal signs of approaching dissolution are present, our means of cure should be administered zealously and unremittingly, and be judiciously selected and applied, according to the symptoms which may supervene.

At this period of disorder, the warm bath; blisters over the abdomen; emollient, mucilaginous, and anodyne enemata; small and frequently repeated doses of Dover's powder; injections of the infusion of ipecacuanha with opium, or of a weak infusion of bark and rhubarb; warm poultices over the abdomen; the use of the diaphoretic mixture already noticed; the infusion of catechu given internally or as an enema; and camphor, with ipecacuanha and opium, are often very serviceable. When there is evidence of morbid secretions and fæcal matters still remaining at this stage of the disease, a full dose of calomel and opium may be given at bed-time, in addition to the employment of some of the above remedies, and followed early in the morning either by a full dose of the compound jalap powder taken in aromatic water, or of rhubarb and calcined magnesia. If either of those seem not to answer the purpose intended, a full dose of castor oil may be substituted for them." 283.

The treatment of hepatic dysentery does not differ, in any material point from that which has been detailed above. There will be more need, however, of impregnating the system with mercury than in the simpler forms, in order to remove the hepatic disease. Upon the subordinate means of relieving particular symptoms in dysentery we need not dwell, as they naturally suggest themselves to every well-informed practitioner.

